# Effect of the biosynthesis of the volatile compound phenylacetaldehyde on chloroplast modifications in tea (*Camellia sinensis*) plants

**DOI:** 10.1093/hr/uhad003

**Published:** 2023-01-11

**Authors:** Lanting Zeng, Xiaochen Zhou, Xiumin Fu, Yilong Hu, Dachuan Gu, Xingliang Hou, Fang Dong, Ziyin Yang

**Affiliations:** Guangdong Provincial Key Laboratory of Applied Botany & Key Laboratory of South China Agricultural Plant Molecular Analysis and Genetic Improvement, South China Botanical Garden, Chinese Academy of Sciences, No. 723 Xingke Road, Tianhe District, Guangzhou 510650, China; South China National Botanical Garden, No. 723 Xingke Road, Tianhe District, Guangzhou 510650, China; University of Chinese Academy of Sciences, No. 19A Yuquan Road, Beijing 100049, China; Guangdong Provincial Key Laboratory of Applied Botany & Key Laboratory of South China Agricultural Plant Molecular Analysis and Genetic Improvement, South China Botanical Garden, Chinese Academy of Sciences, No. 723 Xingke Road, Tianhe District, Guangzhou 510650, China; South China National Botanical Garden, No. 723 Xingke Road, Tianhe District, Guangzhou 510650, China; University of Chinese Academy of Sciences, No. 19A Yuquan Road, Beijing 100049, China; Guangdong Provincial Key Laboratory of Applied Botany & Key Laboratory of South China Agricultural Plant Molecular Analysis and Genetic Improvement, South China Botanical Garden, Chinese Academy of Sciences, No. 723 Xingke Road, Tianhe District, Guangzhou 510650, China; South China National Botanical Garden, No. 723 Xingke Road, Tianhe District, Guangzhou 510650, China; University of Chinese Academy of Sciences, No. 19A Yuquan Road, Beijing 100049, China; Guangdong Provincial Key Laboratory of Applied Botany & Key Laboratory of South China Agricultural Plant Molecular Analysis and Genetic Improvement, South China Botanical Garden, Chinese Academy of Sciences, No. 723 Xingke Road, Tianhe District, Guangzhou 510650, China; South China National Botanical Garden, No. 723 Xingke Road, Tianhe District, Guangzhou 510650, China; University of Chinese Academy of Sciences, No. 19A Yuquan Road, Beijing 100049, China; Guangdong Provincial Key Laboratory of Applied Botany & Key Laboratory of South China Agricultural Plant Molecular Analysis and Genetic Improvement, South China Botanical Garden, Chinese Academy of Sciences, No. 723 Xingke Road, Tianhe District, Guangzhou 510650, China; South China National Botanical Garden, No. 723 Xingke Road, Tianhe District, Guangzhou 510650, China; University of Chinese Academy of Sciences, No. 19A Yuquan Road, Beijing 100049, China; Guangdong Provincial Key Laboratory of Applied Botany & Key Laboratory of South China Agricultural Plant Molecular Analysis and Genetic Improvement, South China Botanical Garden, Chinese Academy of Sciences, No. 723 Xingke Road, Tianhe District, Guangzhou 510650, China; South China National Botanical Garden, No. 723 Xingke Road, Tianhe District, Guangzhou 510650, China; University of Chinese Academy of Sciences, No. 19A Yuquan Road, Beijing 100049, China; Guangdong Food and Drug Vocational College, No. 321 Longdongbei Road, Tianhe District, Guangzhou 510520, China; Guangdong Provincial Key Laboratory of Applied Botany & Key Laboratory of South China Agricultural Plant Molecular Analysis and Genetic Improvement, South China Botanical Garden, Chinese Academy of Sciences, No. 723 Xingke Road, Tianhe District, Guangzhou 510650, China; South China National Botanical Garden, No. 723 Xingke Road, Tianhe District, Guangzhou 510650, China; University of Chinese Academy of Sciences, No. 19A Yuquan Road, Beijing 100049, China

## Abstract

Plant volatile compounds have important physiological and ecological functions. Phenylacetaldehyde (PAld), a volatile phenylpropanoid/benzenoid, accumulates in the leaves of tea (*Camellia sinensis*) plants grown under continuous shading. This study was conducted to determine whether PAld production is correlated with light and to elucidate the physiological functions of PAld in tea plants. Specifically, the upstream mechanism modulating PAld biosynthesis in tea plants under different light conditions as well as the effects of PAld on chloroplast/chlorophyll were investigated. The biosynthesis of PAld was inhibited under light, whereas it was induced in darkness. The structural gene encoding aromatic amino acid aminotransferase 1 (*CsAAAT1*) was expressed at a high level in darkness, consistent with its importance for PAld accumulation. Additionally, the results of a transcriptional activation assay and an electrophoretic mobility shift assay indicated *CsAAAT1* expression was slightly activated by phytochrome-interacting factor 3-2 (CsPIF3-2), which is a light-responsive transcription factor. Furthermore, PAld might promote the excitation of chlorophyll in dark-treated chloroplasts and mediate electron energy transfer in cells. However, the accumulated PAld can degrade chloroplasts and chlorophyll, with potentially detrimental effects on photosynthesis. Moreover, PAld biosynthesis is inhibited in tea leaves by red and blue light, thereby decreasing the adverse effects of PAld on chloroplasts during daytime. In conclusion, the regulated biosynthesis of PAld in tea plants under light and in darkness leads to chloroplast modifications. The results of this study have expanded our understanding of the biosynthesis and functions of volatile phenylpropanoids/benzenoids in tea leaves.

## Introduction

Plants produce volatile compounds as signaling molecules with important ecological functions [1]. Volatile compounds can be divided into the following three main classes on the basis of their biosynthetic pathways: volatile terpenes (VTs), volatile fatty acid derivatives (VFADs), and volatile phenylpropanoids/benzenoids (VPBs) [1]. These compounds can be synthesized and emitted from various plant parts (e.g. vegetative, floral, fruit, and root tissues). The volatile compounds (especially VTs and VFADs) from plant vegetative tissues are mostly involved in responses to specific biotic stresses (e.g. herbivores and pathogens) [2]. The responses of VPBs to biotic stress are generally not as strong and rapid as those of VTs and VFADs. Accordingly, there has been relatively limited research on VPBs in leaf tissues. Many VPBs are important for attracting insects to enhance pollination and seed dispersal. Therefore, previous research on VPB biosynthesis and responses to abiotic stresses focused on flowers and fruits [3]. There are a few reports concerning VPB formation in leaf tissues in response to abiotic factors. For example, VPB contents increase in tea (*Camellia sinensis*) leaves under dark conditions [4, 5]. Thus, we speculated that the accumulation of VPBs in darkness may have some physiological effects in tea leaves. *In vitro* investigations in the 1980s revealed that the VPB phenylacetaldehyde (PAld) affects chlorophyll excited states in extracted plant chloroplasts [6, 7]. These findings compelled us to investigate whether there are some relationships among light, VPBs, and chloroplasts. *Arabidopsis thaliana* and *Nicotiana benthamiana* are model plants, but they either lack or contain minimal amounts of VPBs. Therefore, tea leaves with high VPB contents are useful materials for studying stress response mechanisms and the physiological functions of VPBs under different light conditions [8].

Tea, which is an economically valuable crop worldwide, is cultivated primarily for its leaf tissue. Beverages made from tea leaves are among the top three non-alcoholic beverages because of their health attributes and characteristic flavors. Tea is rich in complex and diverse secondary metabolites [9]. Specialized metabolites in tea leaves are responsible for the representative tea characteristics of food and beverages [9], but they also have important biological functions in tea plants [10–14]. In some cases, analyses of model plants can be used to answer scientific questions regarding cultivated crops. However, there is increasing evidence of several differences in the biosynthesis and stress-related responses of metabolites between model plants and cultivated crops. Thus, secondary metabolite research will need to be conducted using cultivated crops. In tea, VPBs are important aroma components and the main contributors to the floral and fruit aroma properties of tea beverages [8]. In addition, some studies have revealed the physiological functions of volatile compounds during tea plant responses to stress [10–14]. For example, indole, (*E*)-4,8-dimethyl-1,3,7-nonatriene, and (*E*)-nerolidol have important roles related to herbivore resistance. The effects of (*E*)-nerolidol on adaptive responses to cold stress and defenses against pathogens have also been confirmed [12, 14]. Light is a crucial external factor regulating plant growth and development. Plant growth patterns vary substantially during exposures to darkness or light. More specifically, in darkness, seedlings grow rapidly (skotomorphogenesis), enabling them to emerge from the soil. Under light, the longitudinal growth rate of seedlings decreases significantly (photomorphogenesis), which helps to decrease energy consumption and maintain stout stems. Shading is a common light-regulating agronomic practice used for increasing tea quality. In our previous study, shading (nearly dark conditions) resulted in increased VPB contents [4]. Hence, we became interested in the potential relationship between VPBs and light as well as the physiological functions of VPBs in tea plants. Plant growth is regulated by light signal transduction pathways, but the physiological functions of specialized metabolites, such as VPBs, in darkness or under light are unclear, as is how the associated metabolic pathway is regulated under light.

In the present study, we revealed that the PAld content reaches peak levels in darkness and decreases significantly under light. We elucidated the mechanisms controlling PAld biosynthesis under light and in darkness by investigating the synthesis-related genes and transcription factors. The role of PAld accumulated in darkness in dark-treated chloroplasts was also explored. Furthermore, the effect of PAld on chlorophyll and the chloroplast was investigated. The objective of this study was to clarify the upstream mechanism regulating VPB formation in response to abiotic stress, while also revealing the physiological functions of VPBs in plants.

## Results

### Upregulated expression of *CsAAAT1* may be responsible for the accumulation of phenylacetaldehyde in darkness

**Figure 1:**
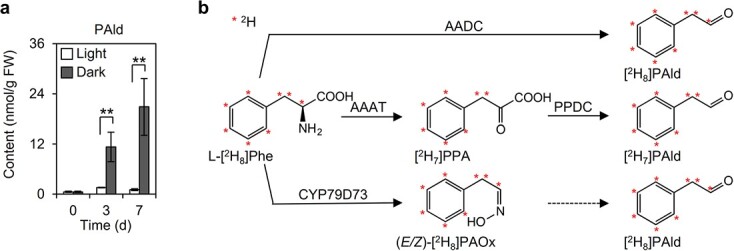
Effects of continuous light and dark treatments on the PAld content in tea leaves and its biosynthetic pathways. **a** PAld content after continuous light and dark treatments. FW, fresh weight. Data are presented as mean ± standard deviation (*n* = 3). ^**^ indicates a significant difference between the two treatments at the same time-point (*P* ≤ .01). **b** Biosynthetic pathways for PAld derived from L-Phe in tea plants. PAOx, phenylacetaldoxime.

**Figure 2:**
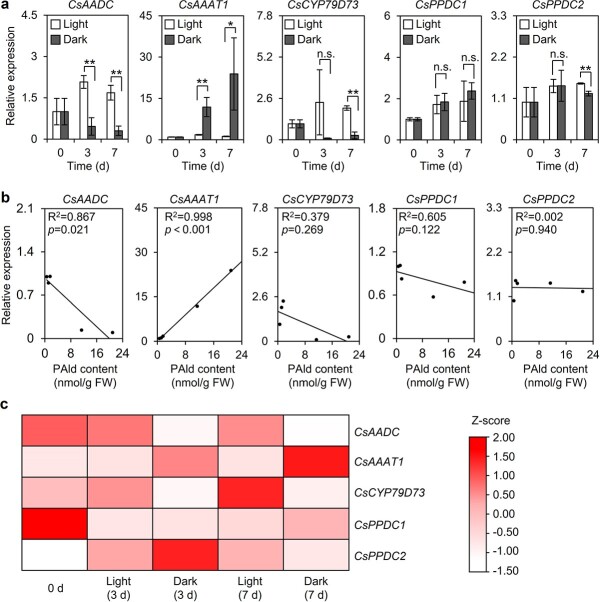
Effects of continuous light and dark treatments on PAld biosynthetic gene expression levels in tea leaves. **a** Expression levels of genes involved in PAld biosynthesis in tea leaves under continuous light and dark treatments. Data are presented as mean ± standard deviation (*n* = 3). ^*^ and ^**^ indicate significant differences between the two treatments at the same time-point (*P* ≤ .05 and *P* ≤ .01, respectively); n.s. indicates there were no significant differences between treatment*s*. **b** Correlations between PAld contents and PAld biosynthetic gene expression levels in tea leaves under continuous light and dark treatments. FW, fresh weight. **c** Heat map of the expression of genes involved in PAld biosynthesis in tea leaves under continuous light and dark treatments. Different rows represent different genes. The color represents the gene expression level under continuous light and dark treatments. The expression levels of the biosynthetic genes were normalized using the *Z*-score formula. 0 d, no treatment; Light (3 d), continuous light treatment for 3 days; Dark (3 d), continuous dark treatment for 3 days; Light (7 d), continuous light treatment for 7 days; Dark (7 d), continuous dark treatment for 7 days.

**Figure 3:**
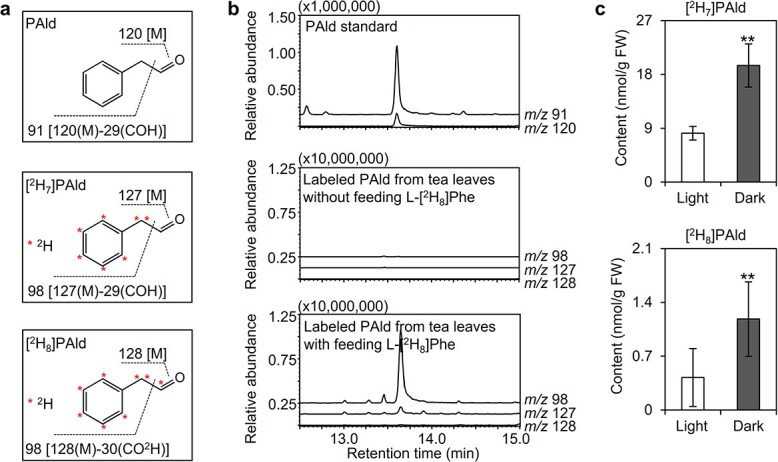
Effect of continuous darkness on the PAld biosynthetic pathway in tea leaves. **a** Characteristic fragment ions and **b** chromatographic separation of labeled PAld from tea leaves fed with L-[^2^H_8_]Phe. **c** Labeled PAld content in tea leaves fed with L-[^2^H_8_]Phe under continuous white light and dark treatments. Tea leaves were collected after a 7-day treatment. FW, fresh weight. Data are presented as mean ± standard deviation (*n* = 4). ^**^ indicates a significant difference between the two treatments (*P* ≤ .01).

Previous research indicated that an exposure to continuous darkness induces PAld accumulation in tea leaves [4, 5]. The findings of the current study were consistent with the results of these earlier studies (Fig. 1a). To clarify why PAld accumulated in darkness, the expression levels of genes responsible for PAld biosynthesis were examined. In tea plants there are three pathways for the biosynthesis of PAld from l-phenylalanine (L-Phe) (Fig. 1b). The related genes include *CsAADC* (aromatic amino acid decarboxylase), *CsAAAT* (aromatic amino acid aminotransferase), *CsPPDC* (phenylpyruvic acid decarboxylase), and *CsCYP79D73* [15]. In the present study, quantitative real-time polymerase chain reaction (qRT–PCR) and RNA-sequencing (RNA-seq) analyses were performed to identify the key genes mediating PAld biosynthesis in darkness. Biosynthetic genes with consistent expression trends revealed by the two analyses were considered further. The *CsAAAT1* expression level increased in darkness, which was in contrast to the decreased *CsAADC* and *CsCYP79D73* expression levels (Fig. 2, Supplementary Data Fig. S1a). The PAld content was positively correlated with the *CsAAAT1* expression level (*R*^2^ = 0.998, *P* < .001), but it was negatively correlated with the *CsAADC* expression level (*R*^2^ = 0.867, *P* = .021) (Fig. 2b). Additionally, in the stable isotope tracing experiment, more [^2^H_7_]PAld formed than [^2^H_8_]PAld (>10-fold difference) in the tea leaves fed with L-[^2^H_8_]Phe and the dark treatment increased the formation of labeled PAld (Fig. 3). These results indicate that the biosynthesis of PAld from L-Phe via phenylpyruvic acid (PPA) may be responsible for the formation of PAld in darkness (Fig. 1b). Therefore, we speculated that the increased PAld content may be attributed to *CsAAAT1* expression in darkness. Although the *CsAADC* and *CsCYP79D73* expression levels were lower in darkness (Fig. 2, Supplementary Data Fig. S1a) than under continuous light, more [^2^H_8_]PAld formed in darkness than under light (Fig. 3c). The increased transformation of (*E/Z*)-[^2^H_8_]PAOx into [^2^H_8_]PAld in a reaction catalyzed by an unknown enzyme in darkness may explain this phenomenon (Fig. 1b), but this possibility will need to be verified in future studies. The *CsPPDC* expression data were inconsistent between the qRT–PCR and RNA-seq analyses (Fig. 2, Supplementary Data Fig. S1a). Accordingly, experiments involving transient expression or mutant plant systems will need to be conducted to functionally characterize *CsPPDC* genes in terms of their potential contribution to PAld formation in darkness.

### CsPIF3-2 might be involved in the regulation of *CsAAAT1* expression

**Figure 4:**
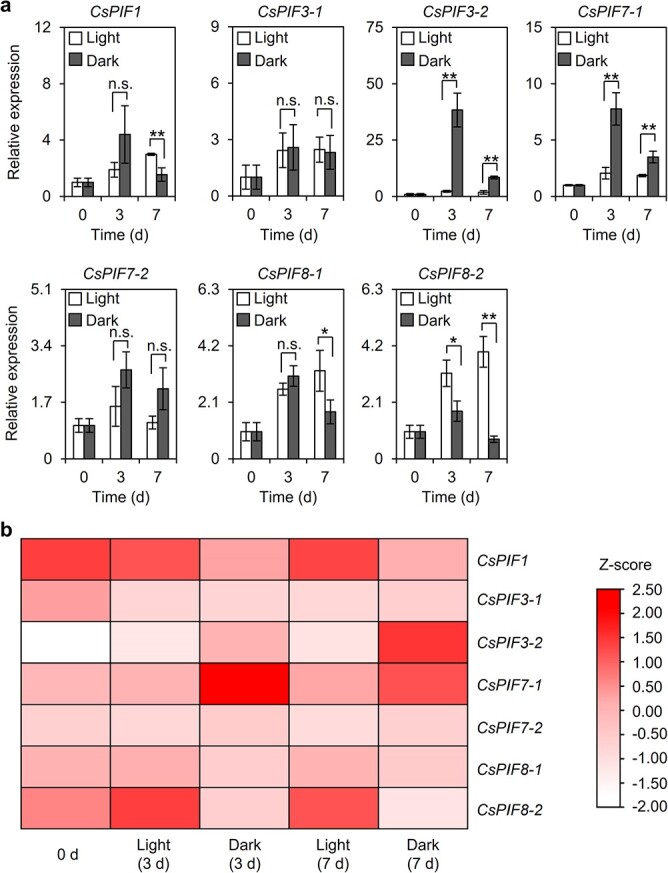
Effects of continuous light and dark treatments on *CsPIF* expression levels in tea leaves. **a***CsPIF* expression levels under continuous white light and dark treatments. Data are presented as mean ± standard deviation (*n* = 3). ^*^ and ^**^ indicate significant differences between two treatments at the same time-point (*P* ≤ .05 and *P* ≤ .01, respectively); n.s. indicates there were no significant differences between the two treatments at the same time-point. **b** Heat map of *CsPIF* expression levels in tea leaves under continuous light and dark treatments. Different rows represent different genes. The color represents the gene expression level under continuous light and dark treatments. *CsPIF* expression levels were normalized using the *Z*-score formula. 0 d, no treatment; Light (3 d), continuous light treatment for 3 days; Dark (3 d), continuous dark treatment for 3 days; Light (7 d), continuous light treatment for 7 days; Dark (7 d), continuous dark treatment for 7 days.

**Figure 5:**
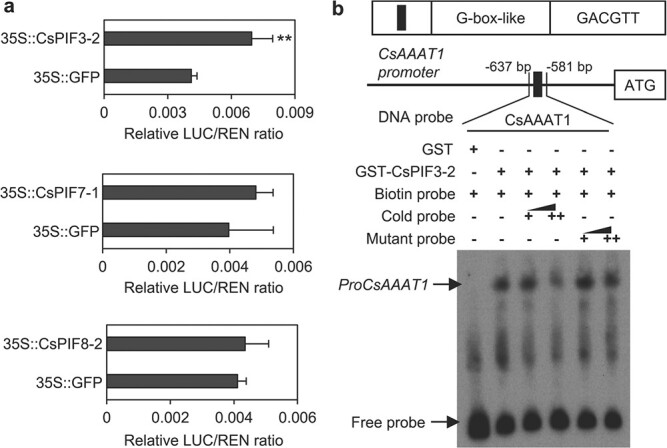
Regulatory effects of CsPIFs on *CsAAAT1* expression under continuous darkness. **a** Analysis of the activation of *CsAAAT1* transcription by CsPIFs in tobacco. Regulation of *CsAAAT1* expression by CsPIFs was determined on the basis of the LUC:REN ratio. Data are presented as mean ± standard deviation (*n* = 3). ^**^ indicates significant differences between CsPIF3-2/7-1/8-2 and the control (35S::GFP) (*P* ≤ .01). **b** Binding of CsPIF3-2 to the G-box-like element in the *CsAAAT1* promoter. Recombinant GST-CsPIF3-2 [GST (glutathione *S*-transferase) as control] was incubated with the biotin-labeled *CsAAAT1* fragments (56 bp). The unlabeled and mutated *CsAAAT1* fragments were used as cold competitors. A gradient concentration of unlabeled and mutant probes was used. The symbols − and + indicate absence and presence, respectively, whereas ++ indicates a 500-fold molar excess of the unlabeled probe and mutant probe relative to the biotin-labeled probe.

Phytochrome-interacting factors (PIFs) have been proposed to regulate plant growth and development in darkness, with a central role in the activation of various structural genes[16]. Some *cis*-elements containing ACGT (named ‘G-box-like’) are reportedly binding sites for PIF transcription factors[17]. We analyzed the *CsAAAT1* promoter region (Supplementary Data Table S1) and detected the G-box-like (GACGTT) sequence (−602 to −607, relative to ATG) (Supplementary Data Fig. S2a). We speculated that PIFs may be involved in the regulation of *CsAAAT1* expression in darkness. On the basis of an analysis of sequence homology, seven *CsPIF* genes were identified in tea [18]. The qRT–PCR and RNA-seq analyses indicated that *CsPIF3-2* and *CsPIF7-1* expression levels increased significantly in darkness, whereas *CsPIF8-2* expression decreased significantly (Fig. 4, Supplementary Data Fig. S1b). To investigate whether CsPIFs can regulate *CsAAAT1* expression, we performed a dual-luciferase reporter assay using tobacco. The *CsPIF3-2*, *CsPIF7-1*, and *CsPIF8-2* open reading frames were cloned into the pEAQ-GFP vector for the production of various effectors, whereas the *CsAAAT1* promoter was inserted into the pGreenII 0800-LUC double-reporter vector to be used as a reporter (Supplementary Data Fig. S2b). We observed that CsPIF3-2 could activate the transcription of *CsAAAT1* in tobacco, but the activation was relatively weak (Fig. 5a, Supplementary Data Fig. S2c). An additional factor may be needed during the activation of *CsAAAT1* expression by CsPIF3-2, which may help to explain the minor effect of CsPIF3-2 alone on *CsAAAT1* transcription. Furthermore, to test whether CsPIF3-2 can bind to the G-box-like sequence in the *CsAAAT1* promoter, we performed an electrophoretic mobility shift assay (EMSA). The EMSA results suggested that CsPIF3-2 can bind to the G-box-like motif in the *CsAAAT1* promoter (Fig. 5b). However, the cold probe did not efficiently compete with the labeled probe for the binding site in this experiment, indicating that the binding of CsPIF3-2 to the G-box-like motif was weak. Because there are multiple PIF-binding sites in the promoter (Supplementary Data Table S1, and Supplementary Data Fig. S2a), CsPIF3-2 may preferentially bind to another *cis*-element in the *CsAAAT1* promoter. Alternatively, it may form a heterodimer with an additional factor, such as another CsPIF, before binding to the promoter, or the PIF requires a post-translational modification before it can bind to the promoter. Therefore, experiments involving mutant plant systems are needed to confirm the regulatory effects of CsPIF3-2 on *CsAAAT1*.

### Phenylacetaldehyde promoted the excitation of chlorophyll in dark-treated chloroplasts

**Figure 6:**
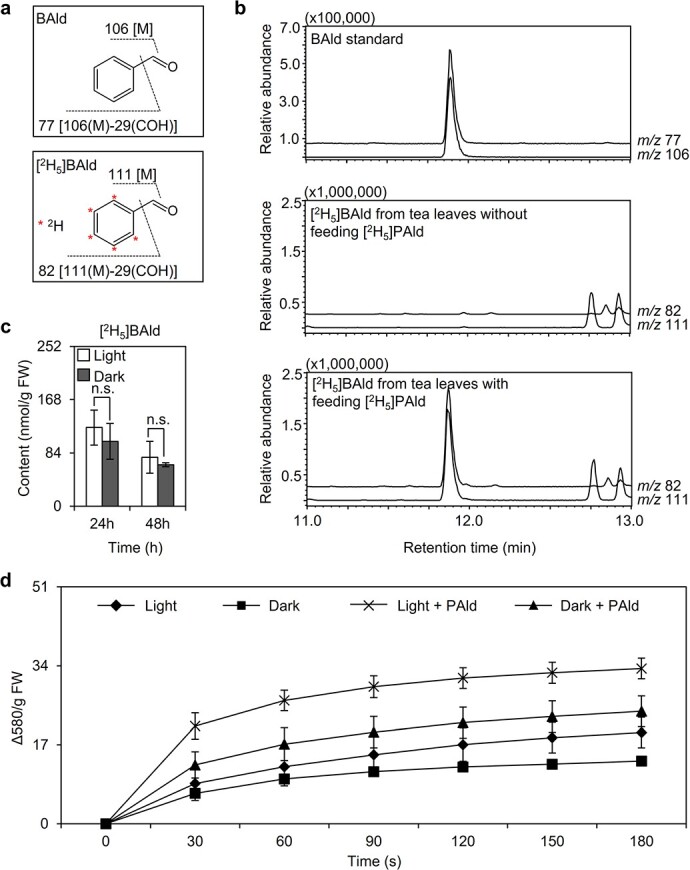
Effect of PAld on the excitation of chlorophyll in dark-treated chloroplasts. **a** Characteristic fragment ions and **b** chromatographic separation of labeled BAld from tea leaves fed with [^2^H_5_]PAld. **c** Labeled BAld content in tea leaves fed with [^2^H_5_]sodium phenylglyoxylate under continuous white light and dark treatments. FW, fresh weight. Data are presented as mean ± standard deviation (*n* = 4). n.s. indicates there were no significant differences between treatments. **d** Reduction of tetrazolium blue promoted by the PAld/chloroplast system.

Earlier research demonstrated that PAld can be converted to benzaldehyde (BAld) and formic acid by plant extracts [6]. In this process, the oxidant is oxygen and the catalyst is peroxidase. The products formed are epoxypentane decomposition intermediates, indicating that electron-excited products may be produced in this process. In this study, [^2^H_5_]BAld was detected in tea leaves treated with [^2^H_5_]sodium phenylglyoxylate (Fig. 6a–c), suggestive of an enzyme-mediated conversion of PAld to BAld in tea plants, possibly leading to the formation of electron-excited products. Researchers previously proposed that in the absence of light PAld can increase the excitation efficiency of chlorophyll in chloroplasts and decrease O_2_ consumption by chloroplasts [6]. This possibility was tested by conducting an *in vitro* experiment, which indicated that PAld added to chloroplasts isolated from tea leaves can enhance the reduction of tetrazolium blue (Fig. 6d). The reduction can be used to reflect the accumulation of O_2_ and a decrease in O_2_ consumption by chloroplasts in the presence of PAld. This phenomenon was observed in chloroplasts that underwent a long-term exposure to darkness and light. These experiments confirmed that PAld can promote the excitation of chlorophyll in dark-treated chloroplasts.

### Phenylacetaldehyde decreased the chlorophyll content and induced chloroplast degradation

**Figure 7:**
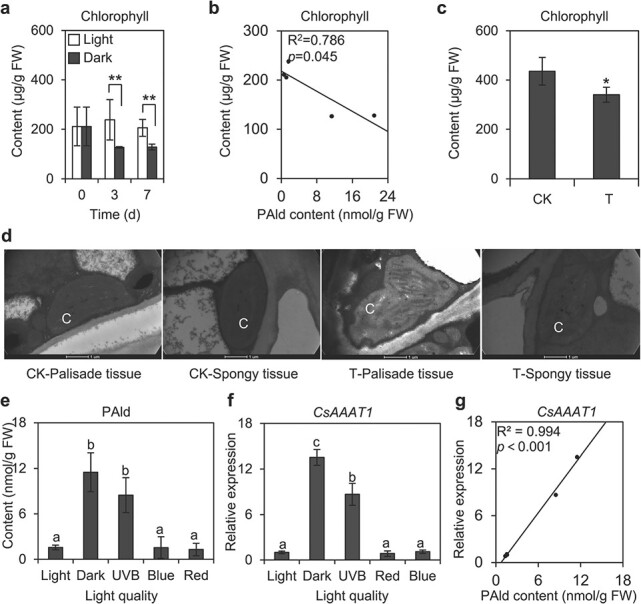
Effect of PAld on chloroplasts and the inhibition of this effect in tea leaves under continuous light. **a** Chlorophyll contents under continuous white light and dark treatments. FW, fresh weight. Data are presented as mean ± standard deviation (*n* = 3). ^**^ indicates a significant differences between the two treatments at the same time-point (*P* ≤ .01). **b** Correlation between the PAld and total chlorophyll contents in tea leaves. **c** Chlorophyll content following an exogenous PAld treatment. CK, tea leaves treated with dichloromethane for 2 days (500 μl every 12 hours for a total of 2 ml); T, tea leaves treated with a PAld standard for 2 days (10 mM dissolved in CH_2_Cl_2_; 500 μl every 12 hours for a total of 20 μmol). ^*^ indicates significant differences between the two treatments (*P* ≤ .05). **d** Chloroplast (C) morphology in tea leaves following an exogenous PAld treatment. **e** PAld content in tea leaves in response to continuous exposure to different light qualities. Data are presented as mean ± standard deviation (*n* = 3). Significantly different mean values are indicated by different letters (*P* ≤ .05). **f** Expression levels of genes involved in PAld biosynthesis in tea leaves exposed to different light qualities. **g** Correlation between PAld content and *CsAAAT1* expression level in response to different light qualities.

The analysis of the chlorophyll content and its correlation with the PAld content indicated that the chlorophyll content decreased in tea leaves exposed to continuous darkness and was negatively correlated with the PAld content in tea leaves (Fig. 7a and b). These results suggest that PAld may be crucial for chlorophyll degradation in darkness, which compelled us to investigate the possible physiological function of PAld in tea plants. Tea leaves treated with exogenous PAld (Supplementary Data Fig. S3) were incubated under continuous white light to inhibit the formation of endogenous PAld. The chlorophyll in the treated tea leaves was analyzed. Interestingly, chlorophyll was significantly degraded by PAld (Fig. 7c). Because PAld is insoluble in water, the PAld standard was dissolved using dichloromethane (CH_2_Cl_2_). Furthermore, the CH_2_Cl_2_ treatment had no significant effects on the chlorophyll content of tea leaves (Supplementary Data Fig. S4). Additionally, self-degradation of chlorophyll was undetectable during the 2-day treatment. The direct effect of PAld on chlorophyll extracted from tea leaves was also investigated. We observed that PAld did not directly affect chlorophyll extracted from tea leaves in 95% ethanol. The results indicated that the degradation of chlorophyll by PAld may be due to the signal transduction regulating chlorophyll metabolism in tea leaves. We also examined chloroplast morphology in tea leaves treated with the PAld standard. Transmission electron microscopy images revealed the chloroplast degradation induced by exogenously applied PAld in tea leaves, with the extent of degradation increasing over time (Fig. 7d). In summary, these findings suggest that PAld has detrimental effects on chlorophyll and chloroplasts. Therefore, PAld formation may be inhibited by white light to maintain photosynthetic activities in tea leaves.

### Phenylacetaldehyde biosynthesis was inhibited under continuous blue and red light

To clarify the mechanism mediating the light-induced inhibition of PAld formation in tea leaves, the PAld content and *CsAAAT1* expression level under different light-quality treatments were analyzed. Both PAld formation and *CsAAAT1* expression were inhibited by blue and red light (Fig. 7e and f). Additionally, the PAld content and the *CsAAAT1* expression level were similarly positively correlated under blue and red light (Fig. 7e–g, Supplementary Data Fig. S1c). Therefore, we speculated that PAld biosynthesis is inhibited under white light because of the exposure to red and blue light (i.e. a major component of visible light). These results suggest that *CsAAAT1* might encode the regulator of the differential accumulation of PAld in tea leaves under white light and in darkness. We also investigated the effects of short-term and diverse light-intensity treatments on PAld formation in tea leaves. After PAld formed in tea leaves following a 4-day exposure to continuous darkness, its production was not rapidly inhibited by light within 8 hours (Supplementary Data Fig. S5a). Accordingly, the light-induced inhibition of PAld formation was not a short-term systemic response to light. Furthermore, compared with the effects of continuous darkness, PAld formation was significantly inhibited by weak blue and red light, and the inhibitory effect was dependent on the light intensity (Supplementary Data Fig. S5b). Notably, major light components, including blue and red light, inhibit PAld biosynthesis by decreasing the *CsAAAT1* expression level, further confirming the role of *CsAAAT1* in PAld biosynthesis.

## Discussion

### Light inhibits *CsAAAT1* expression, which affects one of multiple biosynthetic pathways to decrease the phenylacetaldehyde content

Among VPBs in plants, including tea, 2-phenylethanol (2PE) has been extensively studied because it is one of the main rose-like aroma compounds [15]. In contrast, there has been little research on PAld in tea leaves, likely because of its relatively low abundance. The deamination of L-Phe to *trans*-cinnamic acid (CA) is an upstream step in the VPB biosynthetic pathway [19]. However, the biosynthesis of PAld and 2PE, which are volatile phenylpropanoid-related (C6–C2) compounds, does not involve CA. In plants, 2PE is synthesized from L-Phe *via* PAld in reactions catalyzed by AADC and phenylacetaldehyde reductase (PAR), *via* PPA and PAld in reactions catalyzed by AAAT, PPDC, and PAR, and *via *(*E*/*Z*)-phenylacetaldoxime and PAld in reactions catalyzed by CYP79D73 and PAR (Fig. 1b) [20–22]. In these three pathways, PAld is a direct precursor of 2PE. There are also three pathways for PAld formation in plants, including tea. Plants adapt to environmental changes by producing specialized metabolites through multiple pathways. For example, in rose, an alternative 2PE biosynthetic pathway may be activated in response to seasonal temperature changes [23]. Sun *et al*. summarized the current available information regarding multiple pathways for the biosynthesis of plant volatiles [24]. The variability among these pathways is the result of convergent evolution and a possible consequence of the diversity in the adaptive responses to similar environments [25]. In contrast to VFADs and VTs, VPBs are typically associated with abiotic interactions [8]. The flux into the multiple pathways for the biosynthesis of the VPB compounds PAld and 2PE affected by abiotic stresses may provide insights into the predominant role of specific pathways in response to particular stresses. A recent study indicated that a temperature increase results in increased production of 2PE from L-Phe* via* PPA and PAld in tea leaves [15]. In the present study, we observed that PAld accumulated in darkness, but not under blue and red light (Figs 1a and 7e). Although there are three pathways for PAld formation, only the pathway that converts L-Phe to PAld *via* PPA was activated by darkness. Furthermore, *CsAAAT1* reportedly encodes a protein responsible for the differential accumulation of PAld under different light conditions [20]. The results of investigations on the changes in PAld contents in response to temperature and light reflect the likely importance of AAAT1 during plant adaptations to abiotic factors.

The AAATs are pyridoxal phosphate-dependent enzymes that catalyze the transamination reactions mediating the reversible conversion of aromatic amino acids to the corresponding keto acids [26–28]. Many AAATs often have broad substrate specificities that enable them to produce the precursors of various specialized metabolites in different plants [29]. The spatiotemporal expression of *AAAT* genes is highly correlated with the downstream production of specialized metabolites [30]. Additionally, earlier research demonstrated that *AtTAT1* (Tyr aminotransferase) expression is upregulated by various abiotic stress conditions and coronatine treatments [31]. Several studies indicated that the degradation of some amino acids is crucial under energy-limited conditions during dark-induced senescence in plants [32].

### PIF3-2 may be a transcription factor regulating volatile phenylpropanoid/benzenoid production in response to darkness

Light is an important external signal that regulates plant growth and development through photosynthesis and photomorphogenesis. Plants have evolved different light receptors that sense and respond to changes in environmental light signals. Phytochromes, which are the most important photoreceptors in plants, are modulated by red and far-red light to control photomorphogenic signaling [33]. Earlier studies revealed that PIFs can initiate light signal transduction by directly binding to biologically active phytochromes [34]. There are two known pathways mediating the regulatory effects of light on PIFs in plants. Specifically, PIF protein stability is regulated by activated phytochromes, whereas the expression of PIF transcription factors is controlled by light. The PIF transcription factors belong to a bHLH (basic helix–loop–helix) subfamily and are also known as PILs (phytochrome-interacting factor-like). Moreover, PIFs have a DNA-binding domain that binds to the *cis*-element G-box (CACGTG) in the promoter region of target genes [35]. In darkness, PIFs function as negative regulators of light responses in etiolated seedlings [36].

Eight PIFs have been identified in *A. thaliana* [37], whereas seven PIFs in tea have been reported [18]. Among these proteins, PIF3 was the first PIF family member to be identified [38]. It was subsequently revealed to bind to both phyA and phyB (i.e. phytochromes) through different motifs [35]. It primarily functions as a negative regulator of seedling de-etiolation [36]. Moreover, the regulatory effects of PIF3 on secondary metabolites have been determined. For example, phytochromes regulate anthocyanin biosynthesis *via *signaling pathways involving PIF3, which binds to the promoters of anthocyanin biosynthesis-related genes [39, 40], indicating that light is a crucial regulator of anthocyanin synthesis [41]. Chlorophyll, which is another important secondary metabolite in plants, is also reportedly regulated by PIF3 [42]. More specifically, PIF3 directly interacts with histone deacetylase HDA15 to decrease the acetylation level of chlorophyll biosynthesis-related genes, thereby repressing chlorophyll production in etiolated seedlings [43]. In addition to *A. thaliana*, PIF3 regulates secondary metabolism (mostly related to anthocyanin and chlorophyll) in other plants [44, 45]. In this study, we revealed the effect of CsPIF3-2 on the expression of *CsAAAT1*, which is responsible for PAld biosynthesis in darkness (Fig. 5a, Supplementary Data Fig. S2c), implying PIF3 is important for the biosynthesis of secondary metabolites. However, because of the lack of an efficient genetic transformation system in tea, we were unable to obtain more direct and valid evidence. Therefore, future studies will need to characterize PIF regulatory functions influencing secondary metabolism.

### Phenylacetaldehyde might play a relatively underappreciated role in the excitation of chlorophyll

As early as the 1980s, researchers were exploring chemical- and enzyme-based excitations of electrons (e.g. PAld/peroxidase/O_2_ system) [46], during which BAld and formic acid are formed. Similar to several other enzymatic self-oxidation reactions, the excited state intermediate is a triplet BAld. A previous cellular study confirmed that the activation of this system in the aquatic fungus *Blastocladiella emersonii* leads to lipid peroxidation [7]. The process appears to be induced by an activator that presumably extracts hydrogen from unsaturated fats. The addition of PAld to extracted chloroplasts enhances the excitation of chlorophyll through the transfer of energy from excited species (e.g. triplet BAld) [6]. Hence, PAld might induce the formation of new lipid peroxidation states in the absence of light and participate in the transfer of electron energy within the cell (Fig. 6) [46]. However, PAld formation can lead to the degradation of chloroplasts and chlorophyll (Fig. 7c and d). Thus, under light (i.e. daytime), red and blue light can inhibit the synthesis of PAld, thereby minimizing its adverse effects on chlorophyll and chloroplasts.

In response to stress, plants produce certain volatile metabolites that protect against external stresses. In an earlier study, UVB significantly modulated the proportion of protective terpenes, resulting in an increase in the β-farnesene and α-farnesene contents in *Vitis vinifera* L. and ginseng, respectively [47]. Moreover, UVC-irradiated *A. thaliana* can produce volatiles that function as signaling molecules that may be perceived by tobacco [48]. In addition, (*E,E*)-α-farnesene, β-caryophyllene, limonene, and β-pinene contents may also be affected by temperature and relative humidity [49]. In plants, many stresses usually induce the accumulation of reactive oxygen species (ROS). The volatile metabolite isoprene decreases the harmful effects of oxidative stress by controlling the oxidative state of cells [50]. Furthermore, antioxidants may affect ROS signal transduction or enhance membrane stability. High-temperature stress can decrease the photosynthetic rate in plant leaves, whereas the increased membrane stability induced by isoprene may enhance the tolerance of plants to high temperatures [50]. Studies in maize indicated that the green leaf volatile (*Z*)-3-hexene-1-acetate can protect plants from cold stress, while also restricting phytophagous insect infestations [51]. A similar phenomenon has been observed in tea plants. Under low-temperature conditions, (*E*)-nerolidol can accumulate as a glycoside or as a signaling molecule that stimulates the anti-cold defense mechanism in tea plants, thereby preventing chilling-induced damages [52]. When exposed to drought stress, cells produce volatile metabolites, such as terpenoid metabolites, and lipoxygenase pathway products (e.g. C6 aldehydes, alcohols, and their derivatives) as antioxidants that eliminate excessive amounts of ROS [53]. Most of the related studies focused on stress responses and tolerance. In this study, we revealed the importance of PAld for the excitation of chlorophyll in dark-treated tea plants (Fig. 6).

In this study, the accumulation of PAld was activated in darkness, which was consistent with the high *CsAAAT1* expression level. The results of a transcriptional activation analysis and an EMSA indicated that CsPIF3-2 can slightly and positively regulate *CsAAAT1* expression. The accumulated PAld can be converted to triplet BAld, which affects the excitation of chlorophyll in dark-treated chloroplasts and participates in electron energy transfer in cells. However, an analysis of the effects of exogenous PAld on tea leaves demonstrated that PAld can degrade chloroplasts and chlorophyll. Therefore, under normal light conditions tea plants might use red and blue light to inhibit the synthesis and accumulation of PAld to maintain normal photosynthetic activities. The mechanism underlying light-regulated PAld formation was elucidated. The results of this study will be useful for clarifying the formation of volatile metabolites in tea plants exposed to stress.

## Materials and methods

### Plant materials

The *C. sinensis* cv. ‘Jinxuan’ plants used in this study were grown at the Yingde Tea Experimental Station of the Tea Research Institute, Guangdong Academy of Agricultural Sciences (Yingde, China).

### Light treatments of tea leaves

A previous study demonstrated that tea branches cultivated continuously in water can survive and respond normally to external stresses [15]. In the current study, tea branches were exposed to different light conditions as follows. First, tea branches collected in November 2019 were incubated under continuous white light (6000–6500 lx) or in darkness for 0, 3, and 7 days. Second, tea branches collected in May 2017 were used to investigate the effect of a short-term light treatment. Specifically, tea branches were pretreated in continuous darkness for 4 days. They were then divided into two groups, with one group incubated under continuous white light (6000–6500 lx) and the other group incubated in darkness for 0, 2, 4, and 8 h. Third, tea branches collected in May 2017 were treated with different light qualities or intensities. Briefly, tea branches were incubated under continuous white light (6000–6500 lx), UVB light (1500–2000 lx), weak> blue light (1500–2000 lx), strong blue light (5000–5500 lx), weak red light (1500–2000 lx), or strong red light (5000–5500 lx) for 7 days. Alternatively, they were incubated in darkness for 7 days. All tea branches were maintained at 25 ± 2°C and 70 ± 5% humidity. Tea leaves (second to fourth leaves) were collected from 10–15 branches at each time-point, with three biological replicates per time-point.

### Volatile compound analyses

Volatile compounds were analyzed as previously described [15]. Tea sample extracts were prepared using dichloromethane containing the internal standard ethyl *n*-decanoate. Each extract (1 μl) was analyzed using the QP2010 SE gas chromatography–mass spectrometry system (Shimadzu Corporation, Kyoto, Japan) and the SUPELCOWAX 10 column (30 m × 0.25 mm × 0.25 μm; Supelco Inc., Bellefonte, PA, USA). The mass spectrometer was operated in the full scan mode (*m*/*z* 40–200). The PAld standard was used for the quantitative analysis.

### Stable isotope tracing experiment and determination of labeled products

There are three pathways for the biosynthesis of PAld from L-Phe [15]. To investigate the effect of darkness on the biosynthetic flux, tea branches collected in March 2017 were fed with L-[^2^H_8_]Phe and exposed to different light conditions. Each leaf was treated with 3 μmol/l L-[^2^H_8_]Phe and incubated under continuous white light (6000–6500 lx) or in darkness for 7 days. To test whether PAld was converted to BAld in tea plants, the branches were fed with [^2^H_5_]sodium phenylglyoxylate (500 μl, 6 mM) and incubated under continuous light (6000–6500 lx) for 12 hours. Sodium phenylglyoxylate was used in this study because PAld is insoluble in water. After feeding, tea leaves were incubated under continuous white light (6000–6500 lx) or in darkness for 24 and 48 hours. All tea branches were maintained at 25 ± 2°C and 70 ± 5% humidity. The stable isotope tracing experiment was completed using four biological replicates. The labeled products were determined as described above in the Volatile compound analyses section. The qualitative and quantitative analyses of labeled derivatives were performed on the basis of their unlabeled authentic standards.

### Gene expression analysis

Gene expression was analyzed as previously described [15]. Total RNA was extracted and reverse transcribed into cDNA, which was diluted 40-fold. Gene expression was analyzed by qRT–PCR, which was completed using the LightCycler 480 system (Roche Applied Science, Mannheim, Germany) and the primers listed in Supplementary Data Table S2. The elongation factor 1-α gene (*CsEF-1α*) and *Csβ-actin* were used as the internal reference controls [54]. The specificity of the amplified products was evaluated on the basis of a melting curve analysis. The 2^−ΔΔCt^ method was used to calculate gene expression levels, which were normalized against the expression levels of the internal reference controls.

### Dual-luciferase reporter assay system

The full-length *CsPIF* coding sequences were cloned into pEAQ-GFP-N1 to obtain CsPIF-GFP effector constructs (Supplementary Data Table S3). The empty pEAQ-GFP-N1 plasmid was used as the negative control. The *CsAAAT1* promoter region was amplified by PCR and then inserted into the pGreenII 0800-LUC vector to obtain the reporter construct. Different combinations of the plasmids were used for the *Agrobacterium*-mediated transformation of *N. benthamiana*. Three *N. benthamiana* plants were transformed for each plasmid combination as replicates. After the proteins were expressed, samples were collected and examined using the Dual-Luciferase Reporter Assay System Kit. The results are presented herein as the LUC:REN ratio.

### Electrophoretic mobility shift assay

The EMSA was performed using the LightShift Chemiluminescent EMSA Kit (Thermo Fisher) according to the manufacturer’s instructions. The full-length *CsPIF3-2* coding sequence was cloned into the pGEX4T-3 vector for the expression of the GST-CsPIF3-2 fusion protein (Supplementary Data Table S3). The empty pGEX4T-3 vector was used as the control. After they were expressed in *Escherichia coli* cells, the proteins were purified using glutathione beads. The 56-bp oligonucleotide containing a G-box-like (GACGTT) sequence and a biotin tag (3′-end) was used as the *CsAAAT1* probe. The unlabeled probe and mutant probe (AAAAAA) were used as cold competitors. The probes used for the EMSA are listed in Supplementary Data Table S4.

### Fumigation of leaves with phenylacetaldehyde

Tea branches collected in November 2018 were used to investigate the effect of PAld on the chlorophyll content. Tea branches with tender leaves were incubated under continuous white light at 25 ± 2°C and 70 ± 5% humidity and treated with CH_2_Cl_2_ (500 μl every 12 hours for a total of 2 ml) (CK treatment) or a PAld standard (10 mM dissolved in CH_2_Cl_2_; 500 μl every 12 hours for a total of 20 μmol) [11]. Because of its insolubility in water, PAld was dissolved in CH_2_Cl_2_. Tea branches treated with water were used as the negative controls. The PAld standard and CH_2_Cl_2_ solutions were added to cotton. A diagram representing the investigation of the effect of PAld on tea leaves is provided in Supplementary Data Fig. S3. Samples were collected at 0 hours, 1 day, and 2 days and then examined. Only the fourth leaf was used for the transmission electron microscopy analysis. For each time-point, tea leaves (second to fourth leaves) were collected from 10–15 branches for the chlorophyll analysis, which was performed using five biological replicates.

### Chlorophyll analysis

Chlorophyll contents were analyzed according to a published method [55]. Tea samples were extracted with 80% acetone at 25°C in darkness for 1 day. After centrifuging the samples, the supernatants were collected and measured at 663 and 646 nm using a UV5100 UV-Vis spectrophotometer (Shanghai Metash Instruments Co., Ltd., Shanghai, China). The chlorophyll contents were estimated (mg/g fresh weight) using the following equations: chlorophyll *a* = 12.21 × A_663_–2.81 × A_646_; chlorophyll *b* = 20.13 × A_646_–5.03 × A_663_. The effect of PAld on the chlorophyll contents *in vivo* was also explored. Tea samples were extracted with 95% ethanol at 25°C in darkness for 1 day. After the samples were centrifuged, the supernatants containing chlorophyll were treated with PAld (0, 1, 10, and 100 mM). The chlorophyll contents were measured after the samples were treated for 0, 1, 4, 8, 12, 24, and 48 h.

### Transmission electron microscopy analysis

Tea leaf segments (1 mm × 5 mm) were fixed in 0.1 M phosphate buffer (pH 7.2) containing 2% glutaraldehyde and 2.5% paraformaldehyde. The samples were then fixed in a 1% osmium tetroxide solution. Finally, the fixed samples were dehydrated and embedded in plate molds using EPON 812 resin. After they were cut into sections (80 nm) using a UC7 ultramicrotome (Leica Microsystems GmbH, Wetzlar, Germany), the samples were stained with 4% uranyl acetate and 2% lead citrate. The stained materials were examined using a Tecnai™ G2 Spirit BioTWIN transmission electron microscope (100 kV) (FEI, Hillsboro, OR, USA).

### Extraction of chloroplasts from tea leaves and analysis of the reduction of tetrazolium blue

Two-year-old *C. sinensis* seedlings were incubated under continuous light (~4000 lx) or in darkness at 25 ± 2°C and 70 ± 5% humidity from 23 December 2020 to 5 January 2021. After the treatment, tea leaves were collected (~2 g) and cut into small pieces. Chloroplasts were extracted and the reduction of tetrazolium blue was analyzed as previously described [6]. The leaf pieces were rinsed twice in pre-cooled extraction buffer (pH 7.6) containing 5 mM β-mercaptoethanol, 330 mM sorbitol, 250 mM HEPES, and 0.5 M EDTA. The mixture was blended (1000 rpm) in a cold Waring blender for 10–15 seconds. This step was repeated several times until there were no small pieces remaining. After filtering the solution through one layer of a 20-μm nylon net, the filtrate was centrifuged. The pellet was collected and resuspended in 2.5 ml extraction buffer. The mixture was loaded onto a sucrose gradient (0.5, 0.9, 1.15, 1.35, 1.45, and 1.75 M) and centrifuged. The standard reaction mixtures contained 0.12 M ethanol and 100 μl chloroplast-containing buffer comprising 0.63 M phosphate and 40 mM pyrophosphate. Next, 20 μg PAld was added to the mixture. The final volume and temperature were 1 ml and 25°C, respectively. The reduction of tetrazolium blue was analyzed spectrophotometrically in cells with a 1-cm path length. Specifically, the reduction of the initial dye content (20 μg) was reflected by increases in absorbance at 580 nm.

### RNA extraction and transcriptome sequencing

Total RNA was extracted from tea samples collected in November 2019 (three biological replicates) as described above. The RNA libraries were sequenced using the Illumina NovaSeq 6000 platform. The generated data were analyzed by Bio&Data Biotechnologies Inc. (Guangzhou, China). Clean reads were mapped to the chromosome-scale tea reference genome [56]. HISAT software was used to map the clean reads to the assembled sequences. RSEM software was used to calculate the fragments per kilobase per million reads mapped (FPKM) values, which were used to represent gene expression levels [57, 58]. The values presented in heat maps were normalized using the *Z*-score formula. The clean read data were deposited in the National Genomics Data Center under the accession number PRJCA011336.

### Statistical analysis

A two-tailed Student’s *t*-test completed in Excel was used to determine the significance of the differences between two groups. One-way analysis of variance followed by Duncan’s multiple comparison tests were used to determine the significance of the differences among three or more groups using the SPSS package. A *P* value of ≤.05 was used as the threshold for determining significance.

### Gene accession numbers

The accession numbers of the genes described herein are as follows: *CsAADC*, FS952786; *CsAAAT1*, MH544095; *CsCYP79D73*, XM_028213935.1; *CsPPDC1*, XM_028227140.1; *CsPIF1*, TEA006532; *CsPIF3-1*, TEA033210; *CsPIF3-2*, TEA007077; *CsPIF7-1*, TEA011633; *CsPIF7-2*, TEA025875; *CsPIF8-1*, TEA023842; *CsPIF8-2*, TEA032260.

## Supplementary Material

Web_Material_uhad003

## Data Availability

All relevant data are provided within the paper and its supplementary files. All sequencing data have been uploaded to the National Genomics Data Center (NGDC) under the accession number PRJCA011336.
