# Supplementary material for: Effect of the biosynthesis of the volatile compound phenylacetaldehyde on chloroplast modifications in tea (Camellia sinensis) plants
Source: Hortic Res. 2023 Jan 11;10(3):uhad003. doi: 10.1093/hr/uhad003 (PMC10541522; doi:10.1093/hr/uhad003)
Supplement: Web_Material_uhad003 [file web_material_uhad003.docx]

**Short running title:** Effect of phenylacetaldehyde on the chloroplast

**Title:** Effect of the biosynthesis of the volatile compound phenylacetaldehyde on chloroplast modifications in tea (*Camellia sinensis*) plants

**Authors:** Lanting Zeng ^1, 2, 3 †^, Xiaochen Zhou ^1, 2, 3, †^, Xiumin Fu ^1, 2, 3^, Yilong Hu ^1, 2, 3^, Dachuan Gu ^1, 2, 3^, Xingliang Hou ^1, 2, 3^, Fang Dong ^4^, Ziyin Yang ^1, 2, 3, *^

***Affiliation*:**

*^1^ Guangdong Provincial Key Laboratory of Applied Botany & Key Laboratory of South China Agricultural Plant Molecular Analysis and Genetic Improvement, South China Botanical Garden, Chinese Academy of Sciences, No. 723 Xingke Road, Tianhe District, Guangzhou 510650, China*

*^2^ South China National Botanical Garden, No. 723 Xingke Road, Tianhe District, Guangzhou 510650, China*

*^3^ University of Chinese Academy of Sciences, No.19A Yuquan Road, Beijing 100049, China*

*^4^ Guangdong Food and Drug Vocational College, No. 321 Longdongbei Road, Tianhe District, Guangzhou 510520, China*

^†^ These authors equally contributed to this work.

^*^ **Corresponding authors:** Ziyin Yang, Tel: +86-20-38072989; Email address: zyyang@scbg.ac.cn.

**E-mail addresses:**

Lanting Zeng: zenglanting@scbg.ac.cn; Xiaochen Zhou: zhouxiaochen@scbg.ac.cn; Xiumin Fu: fuxiumin@scbg.ac.cn; Yilong Hu: huyilong@scbg.ac.cn; Dachuan Gu: gudachuan@scbg.ac.cn; Xingliang Hou: houxl@scbg.ac.cn; Fang Dong: dongfangxyz@163.com; Ziyin Yang: zyyang@scbg.ac.cn.

**Supplementary materials**

**Table S1 *cis*-Element in the promoter of aromatic amino acid aminotransferase 1.**

| *cis*-Element | Motif sequence | Start | End |
| --- | --- | --- | --- |
| G-box-like | GACGTT | -602 | -607 |
| Box 4 | ATTAAT | -712 | -717 |
| Box 4 | ATTAAT | -755 | -760 |
| GT1-motif | GTGTGTGAA | -1002 | -1010 |
| MRE | AACCTAA | -1474 | -1480 |
| GT1-motif | GGTTAA | -1477 | -1482 |
| G-box-like | AACGTA | -2054 | -2059 |
| Box 4 | ATTAAT | -2124 | -2129 |
| Box 4 | ATTAAT | -2482 | -2487 |
| Box 4 | ATTAAT | -2865 | -2870 |

**Table S2 Primers used for quantitative real time PCR (qRT-PCR) analysis in the study.**

| Gene | Accession number | Forward primer 5'-3' | Reverse primer 5'-3' |
| --- | --- | --- | --- |
| *CsEF1-α* | KA280301.1 | TTGGACAAGCTCAAGGCTGAACG | ATGGCCAGGAGCATCAAT GACAGT |
| *Csβ-Actin* | HQ420251.1 | GCCATATTTGATTGGAATGG | GGTGCCACAACCTTGATCTT |
| *CsAADC* | FS952786 | GGGAACCTTCAATCTTAC | ACATGCCTTTCTTCTGTC |
| *CsAAAT1* | MH544095 | CGCCGACGAACATCACAATC | CTCTGGGTATGGGAGACCCA |
| *CsCYP79D73* | XM_028213935.1 | GATGATGGCTCGGACGTGTT | CTCATTCAGCCGTGGCTCT |
| *CsPPDC1* | XM_028227140.1 | TGGAATCCGCTGATGCTTACG | GTTTCAGAGGAATGCCTGGA |
| *CsPPDC2* | AB551792.1 | CTTTCAAACCGTTACTTGCTAC | CCTCCAAACCCATCTGATTA |
| *CsPIF1* | TEA006532 | GAGCCAAAACCAGAGATCCA | TAGTATGGGCGAGAGGATCG |
| *CsPIF3-1* | TEA033210 | GAAGTGATGGCGGGTAAGAA | CAGCTGCCACTGATTTTGAA |
| *CsPIF3-2* | TEA007077 | ATGGCTAAGCGACTTGAGGA | CTATTGCCAGAACACGCAGA |
| *CsPIF7-1* | TEA011633 | CGCTGGAATCCATAGTCCAT | ATTCTGACCACATCCGCTTC |
| *CsPIF7-2* | TEA025875 | AAACAGATGCGGTCGAAATC | TCTCGCTCTTCATCCAGGTT |
| *CsPIF8-1* | TEA023842 | GCTGCCTTCATGCCTCTAAC | CAAGGCTGCCATCCTGTTAT |
| *CsPIF8-2* | TEA032260 | TGACCTTGCAGCAACAACTC | CAAGAAGGTAGGGGCATGAA |

*EF1-α*, encoding elongation factor 1; *AADC*, aromatic amino acid decarboxylase; *AAAT1*, aromatic amino acid aminotransferase 1; *PPDC*, phenylpyruvic acid decarboxylase; *PIF*, phytochrome interacting factor.

**Table S3 Primers used for gene cloning in the study.**

| Vector | Forward primer 5'-3' | Reverse primer 5'-3' |
| --- | --- | --- |
| *CsPIF3-2-GFP* | CAAATTCGCGACCGGTATGCCTTTCTCAGAG | TGCTAGTCATACCGGTGCTACTGGCATCAGC |
| *CsPIF7-1-GFP* | CAAATTCGCGACCGGTATGAAAGGAATCATG | TGCTAGTCATACCGGTACCCCCTTGAACATG |
| *CsPIF8-2-GFP* | CAAATTCGCGACCGGTATGAGCCTGTGTGTTCCAAG | TGCTAGTCATACCGGTAGTCTTAGAAGTAGAAGGTG |
| *CsAAAT1promoter-LUC* | GACGGTATCGATAAGCTTACATTTTTTTAATCAAATTA | TCTAGAACTAGTGGATCCCTTTCTTTATGTTTTTGGCG |
| *GST-CsPIF3-2* | GTGGATCCCCGAATTCCATGCCTTTCTCAGAGTTTTA | AGTCGACCCGGGAATTCTCAGCTACTGGCATCAGCAT |

*PIF*, phytochrome interacting factor; *AAAT1*, aromatic amino acid aminotransferase 1.

**Table S4 Probe sequences of electrophoretic mobility shift assay.**

| Probe name | Sequence 5'-3' |
| --- | --- |
| Biotin probe | TGATAAAAGTAGGTTCGTAATGTTATTATTTCAA**GACGTT**TTTCATTTTCTTTTAA |
| Cold probe | TGATAAAAGTAGGTTCGTAATGTTATTATTTCAA**GACGTT**TTTCATTTTCTTTTAA |
| Mutant probe | TGATAAAAGTAGGTTCGTAATGTTATTATTTCAAaaaaaaTTTCATTTTCTTTTAA |


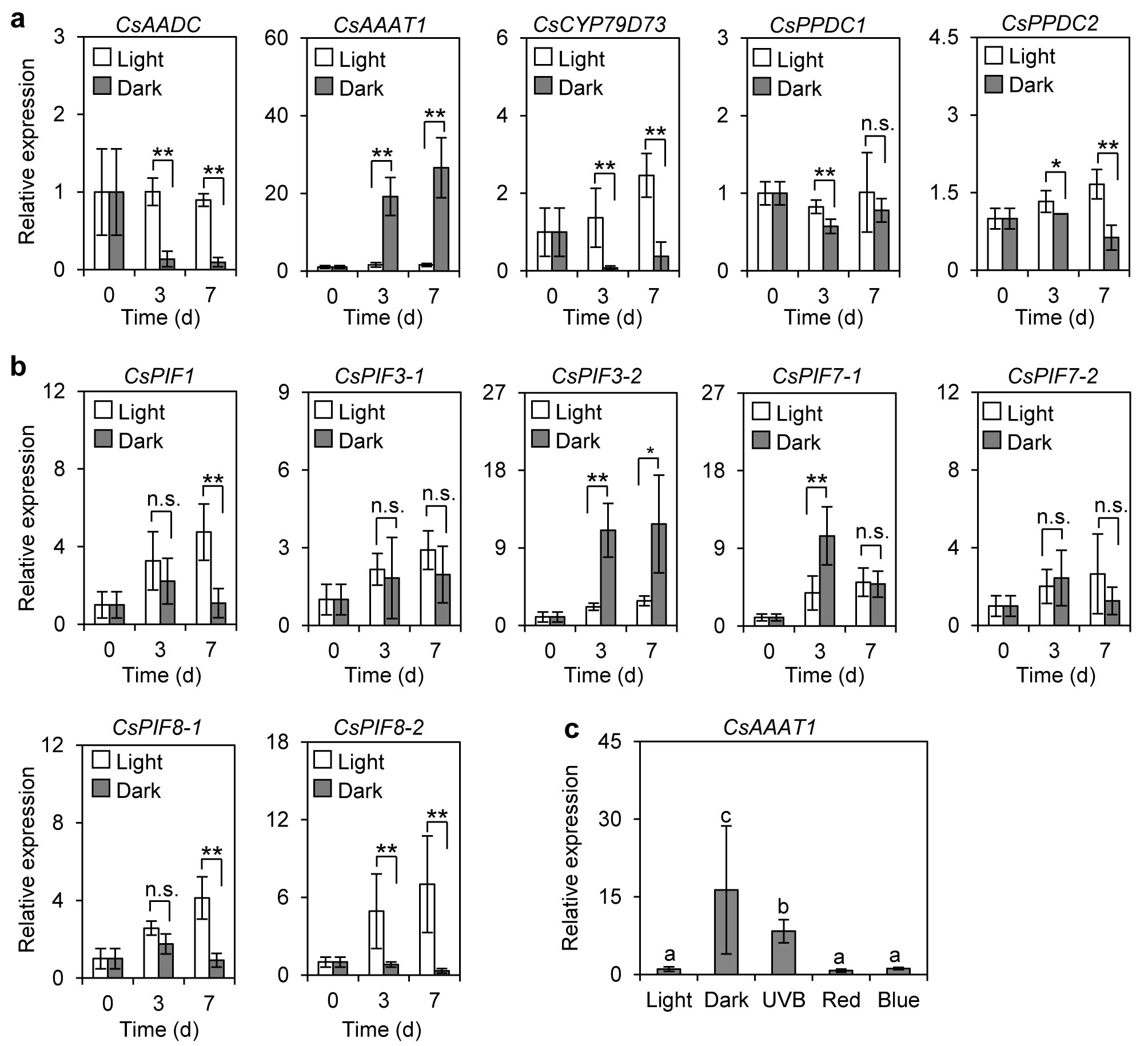


**Fig. S1 Analysis of expression level of genes using *Csβ-actin* as a reference gene in the study.**

(a) Expression levels of genes involved in PAld biosynthesis in tea leaves under continuous light and dark treatments. *AADC*, aromatic amino acid decarboxylase; *AAAT1*, aromatic amino acid aminotransferase 1; *PPDC*, phenylpyruvic acid decarboxylase. (b) *CsPIF* expression levels under continuous white light and dark treatments. *PIF*, phytochrome interacting factor. (c) Expression levels of genes involved in PAld biosynthesis in tea leaves exposed to different light qualities. Data are presented as the mean ± standard deviation (n = 3). (a & b) * and ** indicate significant differences between two treatments at the same time-point (*p* ≤ 0.05 and *p* ≤ 0.01, respectively). n.s. indicates there were no significant differences between two treatments at the same time-point. (c) Significantly different mean values are indicated by different letters (*p* ≤ 0.05).


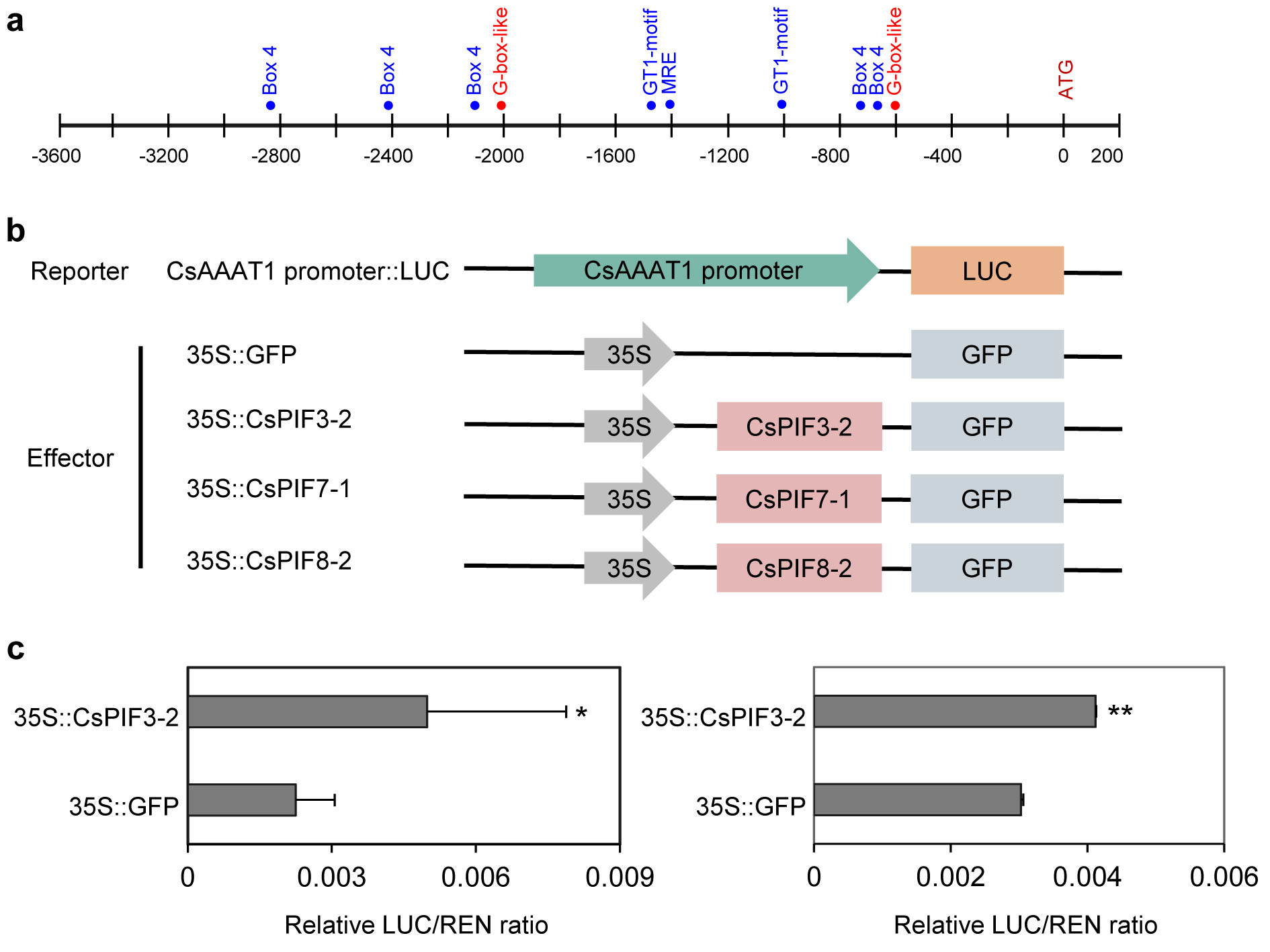


**Fig. S2** **Transcriptional activation analysis of *CsAAAT1* by CsPIF3-2.**

(a) *cis*-Element analysis of *CsAAAT1* promoter. *AAAT1*, aromatic amino acid aminotransferase 1. (b) The diagrams of the double reporters and effector plasmids used in the assay. PIF, phytochrome interacting factor. The reporter and effector vectors were co-introduced into tobacco leaves using *Agrobacterium* GV3101. (c) Analysis of the activation of *CsAAAT1* transcription by CsPIFs in tobacco in other batches. The regulation of *CsAAAT1* expression by CsPIFs was determined on the basis of the LUC-to-REN ratio. Data are presented as the mean ± standard deviation (n = 3). * and ** indicate significant differences between two treatments at the same time-point (*p* ≤ 0.05 and *p* ≤ 0.01, respectively).

**
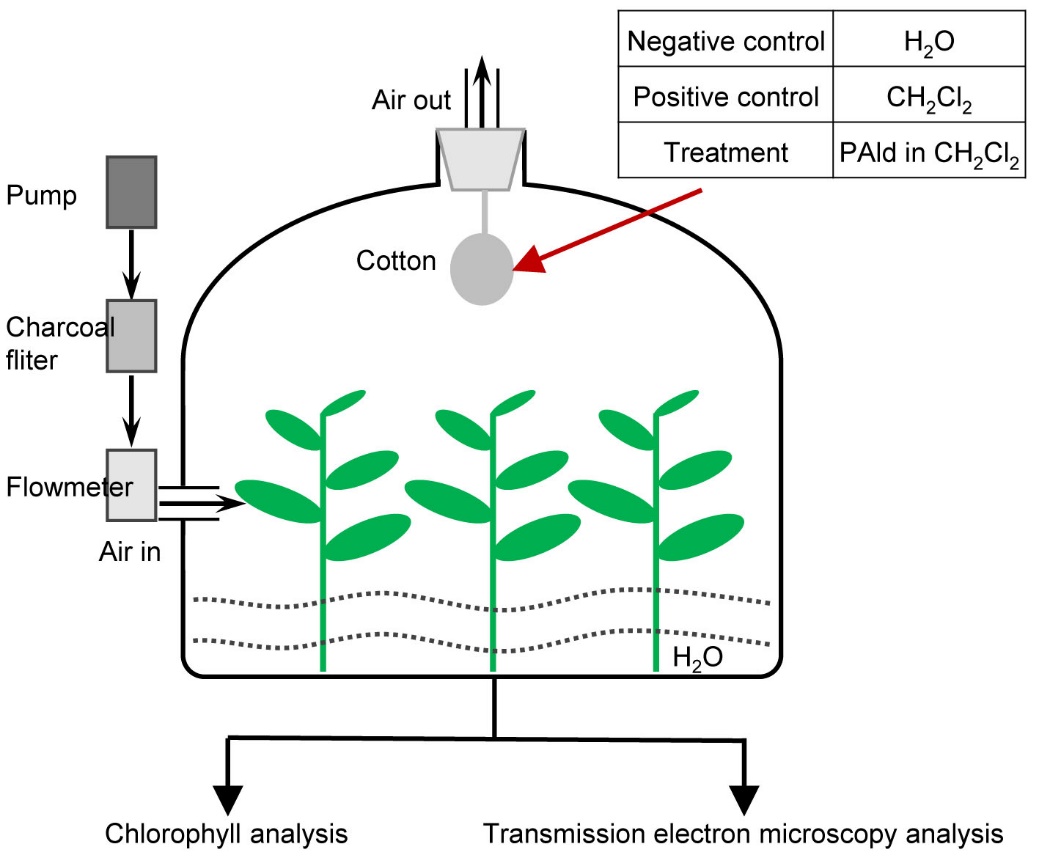
**

**Fig. S3 Experimental design for investigating effect of PAld on tea leaves.**

The black arrow indicates the airflow direction. The air was circulated using a pump. The intake air was filtered through a short plug of charcoal, and airflow was controlled using a flowmeter. CH_2_Cl_2_, dichloromethane; PAld, phenylacetaldehyde.

**
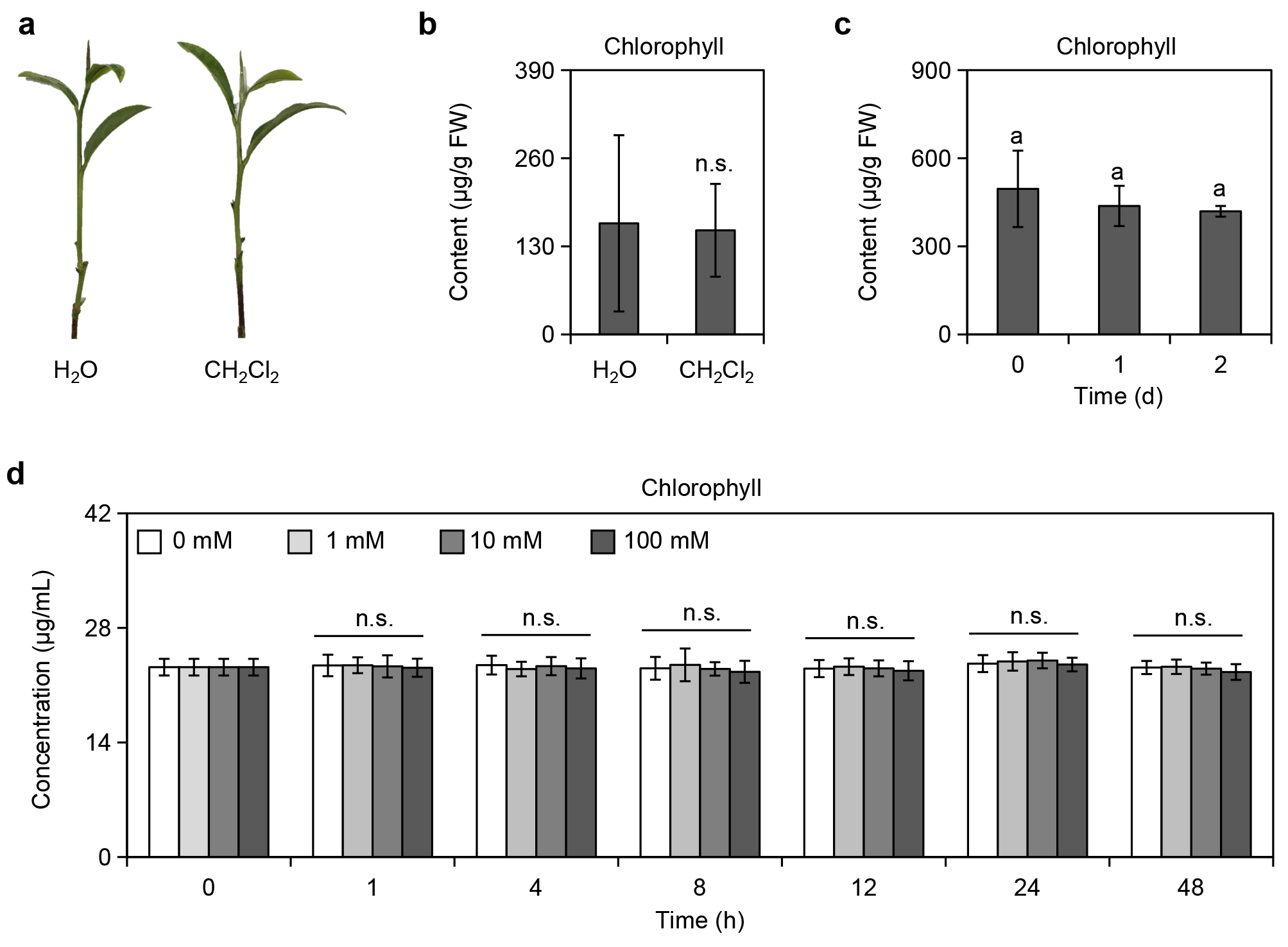
**

**Fig. S4 Effect of dichloromethane (CH_2_Cl_2_) and direct effect of phenylacetaldehyde (PAld) on chlorophyll content in tea leaves.**

(a) Morphology of tea branches after exogenous CH_2_Cl_2_ treatment. (b) Effect of exogenous CH_2_Cl_2_ on chlorophyll content in tea leaves. Tea leaves were treated with CH_2_Cl_2_ for 0 d, 1 d and 2 d. FW, fresh weight. n.s. indicates there were no significant differences between two treatments. (c) Change in content of chlorophyll after extracted from tea leaves. Mean values with the same letter are not significantly different. (d) Direct effect of PAld on chlorophyll content. 0 mM, 1 mM, 10 mM, and 100 mM represent different PAld concentrations in the 95% ethanol containing chlorophyll from tea leaves. Data are presented as the mean ± standard deviation (n=4). n.s. indicates there were no significant differences among the treatments at the same time-point.

**
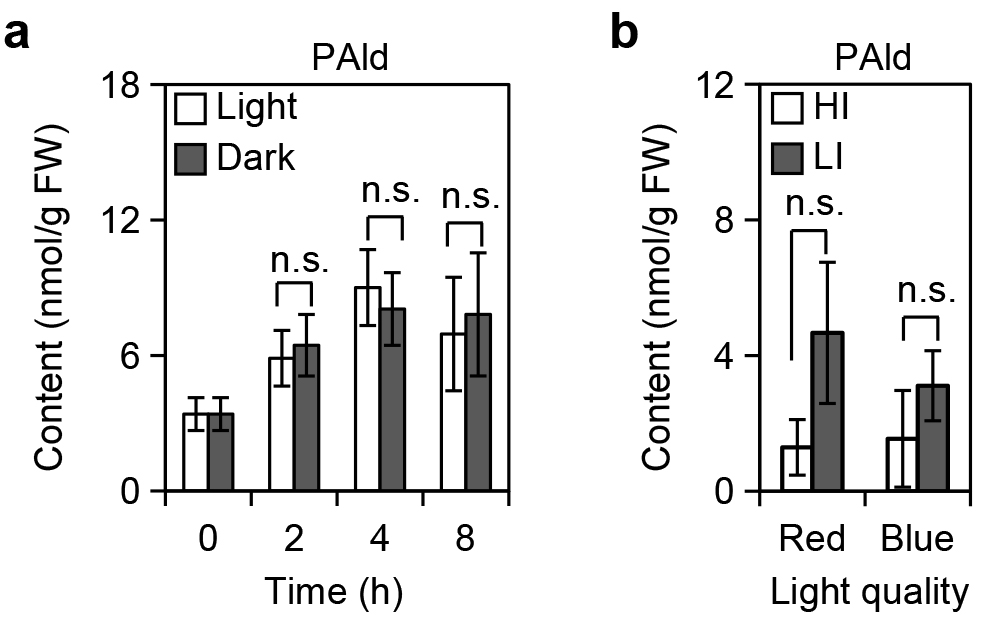
**

**Fig. S5 Effect of short-term and different light intensity treatments on PAld in tea leaves.**

(a) PAld content in tea leaves under short term treatment of white light and dark. Before the short-term treatment of white light and dark, tea branches were cultivated in continuous dark treatment for 4 d. FW, fresh weight; PAld, phenylacetaldehyde. (b) PAld content in tea leaves under different light intensity treatment. Tea leaves were collected after treatment for 7 d. Data are presented as the mean ± standard deviation (n=3). n.s. indicates there were no significant differences between treatments. HI, high intensity; LI, low intensity.
